# Nullomers: Really a Matter of Natural Selection?

**DOI:** 10.1371/journal.pone.0001022

**Published:** 2007-10-10

**Authors:** Claudia Acquisti, George Poste, David Curtiss, Sudhir Kumar

**Affiliations:** 1 Center for Evolutionary Functional Genomics, Arizona State University, Tempe, Arizona, United States of America; 2 The Biodesign Institute and the School of Life Sciences, Arizona State University, Tempe, Arizona, United States of America; University of Maryland, United States of America

## Abstract

**Background:**

Nullomers are short DNA sequences that are absent from the genomes of humans and other species. Assuming that nullomers are the signatures of natural selection against deleterious sequences in humans, the use of nullomers in drug target identification, pesticide development, environmental monitoring, and forensic applications has been envisioned.

**Results:**

Here, we show that the hypermutability of CpG dinucleotides, rather than the natural selection against the nullomer sequences, is likely the reason for the phenomenal event of short sequence motifs becoming nullomers. Furthermore, many reported human nullomers differ by only one nucleotide, which reinforces the role of mutation in the evolution of the constellation of nullomers in populations and species. The known nullomers in chimpanzee, cow, dog, and mouse genomes show patterns that are consistent with those seen in humans.

**Conclusions:**

The role of mutations, instead of selection, in generating nullomers cast doubt on the utility of nullomers in many envisioned applications, because of their dependence on the role of lethal selection on the origin of nullomers.

## Introduction

With the number of completely sequenced genomes approaching a landmark of 1000, it is becoming possible to look for similarities and differences between genomes to make evolutionary and functional inferences. For instance, using the complete genome sequences, investigators are now identifying short sequences that are missing from one or more genomes [1]–[3]. The discovery of the absence of very short sequences (called nullomers) is rather unexpected when we consider that the genomes of higher organisms are extremely large, with a majority consisting of non-coding and non-repetitive sequences [4]. For example, over half of the haploid human genome (∼1.5 billion base pairs) is occupied by unique sequences with no known function, and one would expect to see all possible sequences of up to a length of ∼15 in the human genome.

However, many 11 bp sequence motifs were found to be missing from the human genome [1]. Immediately, these nullomers have been assumed to be caused by the act of negative selection, and thus considered useful for a variety of basic science and application scenarios, including drug target identification, pesticide development, environmental monitoring, and forensics [1], [5]. Is the natural selection against nullomers really the primary cause of their absence in our genomes?

## Analysis and Discussion

We examined whether the well-known mutational characteristics of the human genome may create the observed deficit of some short sequence motifs. At the outset, we observed that ∼50% of the nucleotides in the 80 reported human nullomers of length 11 [1] participate in the CpG dinucleotides (CpGs). This number is 40 times that seen for nucleotides in the non-coding regions of the human genome [6].

**Figure 1:**
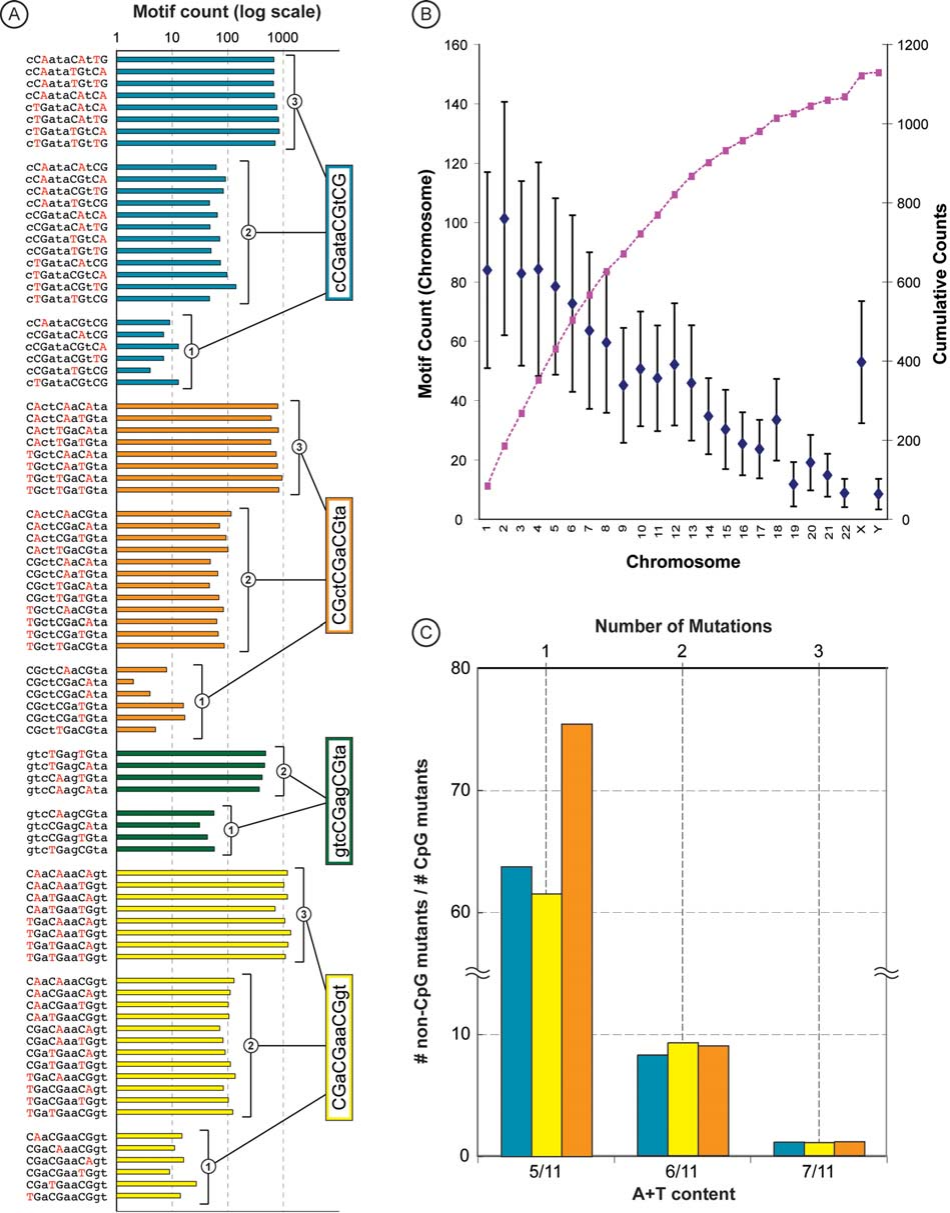
Frequencies of mutated forms of the nullomers in the human genome. (A) The counts of CpG-mutated forms of nullomers (with one, two, and three CpGs mutated to TpG or CpA) found in the non-repetitive portion of the human genome sequence (http://hgdownload.cse.ucsc.edu/goldenPath/hg18/bigZips). The results are shown for the four never-found nullomers in the human genome and polymorphic sequences databases (see Table 3 in ref. [1]). (B) Chromosomal means and standard deviations of the numbers of fully-CpG-mutated forms of the 76 nullomers, which occur with low frequency [1]. Estimates were derived by randomly mutating all CpG positions to either CpA or TpG for each nullomers and then scanning each human chromosome to find their frequency. The set of mutants analyzed contained 76 sequences, because each nullomer contributed one mutated form to the set . (C) The ratio of the average counts of the original and CpG-mutated nullomer sequences. Results are shown for one, two, and three mutations for all nullomers that contain three CpGs (see panel A). Original and CpG mutants with the same A+T content were compared.

A deficit of sequence motifs containing CpGs in vertebrates' genomes has been known for over four decades [7]; it is caused by the hypermutability of CpGs, which mutate at a rate 10–20 times higher than the other point mutations [e.g.8], [9]. Because all reported human nullomers contain multiple CpGs, their absence from the human genome may be caused by the hyper mutation of positions involved in CpG dinucleotides. In this case, we expect to see many 11 bp sequences that show C→T and G→A differences from nullomer sequences, which correspond to the CpG → TpG and CpG → CpA mutations, respectively. This prediction is confirmed by the analysis of the human genome (Fig. 1A, B). In fact, motifs that differ in one, two and three base pairs from the four never-found human nullomers occur with increasing frequencies consistent with the effects of CpG hypermutability. Nullomer-alternatives with all the CpGs mutated occur with the greatest frequencies, and there is an exponential negative relationship between the number of CpGs contained in a motif and its frequency in the human genome (Fig. 1A).

The nullomer-alternatives with one, two, and three CpG mutations contain increasingly larger A+T content, because of C→T and G→A changes in the nullomer mutants. Therefore, we investigated whether the increasing A+T content of the CpG mutated nullomers may cause the trend seen in Fig. 1A, because A+T-rich motifs are more abundant in the genome. For making valid comparisons within the context of the nullomer sequences, we computed the average number of motifs occurrences where all the CpGs in the nullomer sequence were replaced by dinucleotides other than CpA and TpG (non-CpG mutants). We found the ratios of the non-CpG mutants to the CpG mutants for each A+T content category to be close to one when all CpG dinucleotides are mutated, but it becomes 60 times higher when only one CpG is mutated (Fig. 1C). This trend suggests that hypermutability of CpGs is the primary factor responsible for causing a deficit of motifs, which are identified as nullomers.

If CpG hypermutability is a major factor in causing nullomers, we expect to see an overabundance of CpG dinucleotides in the nullomers found in other mammalian genomes. This is indeed the case, as over 50% base pairs in the nullomers identified in chimpanzee, mouse, dog, and cow, genomes are involved in CpGs (Table 1). Interestingly, out of a total of 530 nullomers, not a single one is CpG-free (Table 1). In fact, all nullomers contain at least two CpG dinucleotides (Table 1).

**Table 1:** Proponderance of CpG dinucleotides in the mammalian nullomers of length 11 base pairs.

Species	Nullomers		No. of nullomers with CpGs				
	Count	%CpGs	0	1	2	3	4
*Homo sapiens*	80	53.63	0	0	6	72	2
*Pan troglodytes*	136	54.28	0	0	4	130	2
*Bos taurus*	96	54.55	0	0	4	88	4
*Canis familiaris*	40	54.55	0	0	0	40	0
*Mus musculus*	178	55.36	0	0	0	170	8

Note.–The 11 base pair nullomer sequences were obtained from web resource http://trac.boisestate.edu/dna/applets/SeqCount.html ([1]).

**Figure 2:**
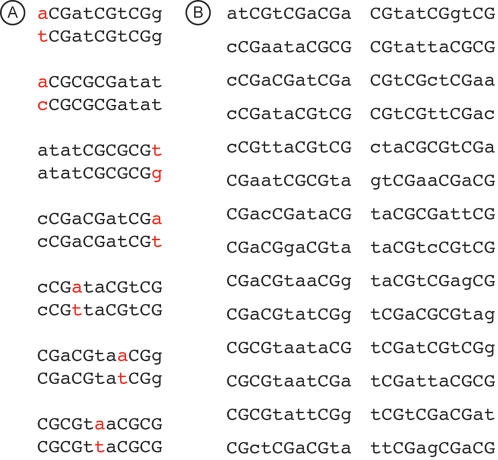
Similarities and differences in human and chimpanzee nullomers. (A) Seven pairs of human nullomers that differ in only one nucleotide. Note that several nullomers are listed twice in ref. 1, as some sequences are reverse complement of others (e.g., atatCGCGCGt and aCGCGCGatat). (B) Set of nullomers that are found in both human and chimpanzee genomes (data from http://trac.boisestate.edu/dna/applets/SeqCount.html) [1].

In addition to hypermutable CpG dinucleotides, regular point mutations appear to have played an important role in generating the observed constellation of human nullomers. This is clearly evident from the comparative sequence analysis of nullomers within human and between human and chimpanzee genomes (Fig. 2). Within humans, 14 out of 80 reported nullomers differ in only one base pair. In addition, humans share 28 nullomers with chimpanzees, and 14 human nullomers differ in one base pair from chimpanzee nullomers. This means that the human set contains nullomers inherited from the common ancestor of human and chimpanzee, in addition to those that have arisen within the human lineage. Furthermore, the human genome shares more nullomers with its closest evolutionary relative chimpanzee than with distantly-related mammals, including mouse (two), cow (none) and dog (none).

In summary, our results suggest that the collection of human and other mammalian nullomers are likely a by-product of the mutational characteristics of the genome, with hypermutable CpG dinucleotides playing a major role. This means that similarities in the mutational patterns among species will lead to the discovery of similar (or common) sets of nullomers among species. Therefore, mutational characteristics, rather than selection pressures, lead to the rare occurrence and absence of certain motifs. This fact will adversely impact envisioned uses of these nullomers, which are contingent on the presupposition that lethality of nullomer sequences to the host individuals is the primary cause of their rarity in our genomes [e.g.1], [5].
